# Protective Effects of Rhubarb in Rats with Acute Pancreatitis and the Role of Its Active Compound Rhein on Mitochondria of Exocrine Cells

**DOI:** 10.1155/2018/7321352

**Published:** 2018-07-22

**Authors:** Jianlei Zhao, George Li, Wenbi Xiong, Litao Liu, Jin Xiang, Mei Tang, Zhu Yuan

**Affiliations:** ^1^Department of Pharmacology, West China School of Basic Medical Sciences & Forensic Medicine, Sichuan University, Chengdu 610041, China; ^2^Faculty of Pharmacy, University of Sydney, NSW 2006, Australia; ^3^Department of Clinical Pharmacology, West China Hospital, Sichuan University, Chengdu 610041, China; ^4^Laboratory of Tumor Biology and Cancer Center, West China Hospital, Sichuan University, Chengdu 610041, China

## Abstract

Da-Cheng-Qi-Decoction (DCQD) has been used in the treatment of acute pancreatitis (AP) in China for many years. The aim of the current study was to examine the principal ingredient rhubarb of DCQD and its potential link to the pancreatic repair effects in rats with AP. The pancreatitis was induced in SD rats by intraperitoneal injections of cerulein. The results showed that rhubarb significantly increased blood perfusion of pancreatic tissue, reversed mitochondrial damage, and promoted pancreatic acinar and stellate cell proliferation. In addition, the rhein (from rhubarb) had high distribution in pancreas tissue and protected mitochondria in AR42J cells via the activation of PI3K/AKT/mTOR signaling pathway and activity inhibition of AMPK (P < 0.05). The results provide some preclinical evidence on the protective effects of DCQD for the treatment of acute pancreatitis. Rhein is regarded to be the active compound of rhubarb and can be expected to be a new compound for the treatment of AP.

## 1. Introduction

Severe acute pancreatitis (SAP) is a very serious systemic disease without any effective treatments. In patients with pancreatic necrosis, about 1/3~1/2 patients will present with diabetes mellitus or transit to chronic pancreatitis after SAP resolves [1, 2]. Investigations that target and promote pancreatic exocrine cells repair in SAP are in great need. Unfortunately, there are no effective western clinical drugs to prevent the emergence of SAP or promote pancreatic regeneration after necrosis [3]. We previously reported that Da-Cheng-Qi-Decoction (DCQD) exerted a significant effect in reducing complications in patients with SAP [4, 5]. Patients usually presented with a reduction in the severity of the condition and a quicker normalization of pancreatic biochemical markers and morphology verified by CT after treatment with DCQD [6, 7]. It markedly reduced pseudocyst formation and promoted the absorption of pancreatic ascites during pancreatic recovery [8, 9]. All these clinical phenomena indicate that DCQD can effectively improve pancreatic regeneration, although the active components of the formula DCQD were not clear.

DCQD is composed of rhubarb (Rheum officinale Baill.), Houpu (Magnolia officinalis Rehd), Zhishi (Fructus Aurantii Immaturus), and Mangxiao (Natrii Sulfas). Rhubarb is the principal ingredient in DCQD, exerting the major therapeutic activity of the decoction. It has been established that a systemic inflammatory response and disturbance of the pancreatic microcirculation occur at almost the same time in SAP and the process of regeneration [10, 11]. In traditional Chinese Medicine (TCM), stewed rhubarb is effective in “activating circulation” [12, 13]. The pancreas distribution characters of the five main anthraquinone compounds of rhubarb (rhein, emodin, aloe-emodin, chrysophanol, and rheochrysidin) were studied in SAP rats treated with DCQD and rhubarb [14, 15]. In both studies, rhein was the most abundant constituent detected in pancreatic tissues among all the anthraquinones. Therefore, the aim of the current study was to examine the protective effects of rhubarb and rhein in rats with acute pancreatitis and exocrine cells.

## 2. Materials and Methods

### 2.1. Reagents

Pure emodin, aloe-emodin, rhein, and chrysophanol, all of which are present in stewed rhubarb, as well as >99% pure zaltoprofen as an internal standard, were purchased from the National Institute for the Control of Pharmaceutical and Biological Products (Beijing, China). HPLC-grade methanol was obtained from Tedia (Ohio, USA). Acetic acid and ethyl acetate were purchased from Chongqing Chemistry Co. Ltd. (Chongqing, China). Analytical-grade ammonium acetate, sodium hydroxide, and hydrochloric acid were purchased from Chengdu Kelong Chemical Reagent Factory (Chengdu, China). All other reagents were obtained from Sigma (New Jersey, USA).

### 2.2. Preparation of Rhubarb Solution

Rhubarb (stewed) granules were purchased from Chengdu Green Herbal Pharmaceutical Co. Ltd. (Chengdu, China). Extraction yield was approximately 5% (w/w, dried extract/crude herb). Before being orally administered to rats, the stewed rhubarb granules were reconstituted with water to a concentration of 1g/ml. The specimens used in this study were stored in Voucher specimens of rhubarb number 2013-0104 and deposited in the Laboratory of Pharmacology, Sichuan University.

### 2.3. Quantitative Analysis of Marker Compounds in Rhubarb

Rhubarb granules were reconstituted in distilled water to a concentration of 6 mg/ml. The mixture was centrifuged at 3000 rpm for 10 min, and 10*μ*l of supernatant was subjected to high performance liquid chromatography (HPLC) analysis. All separations were performed on a Waters HPLC system equipped with a binary pump, autosampler, and Waters 2487 dual-wavelength absorbance detector (Waters, USA). An RP-C18 HPLC column (150 × 4.6 mm, S-5*µ*m, 12nm) and a guard column (GL Sciences, Japan) were used. The mobile phase was a mixture of 100% methanol (solvent A) and H_2_O (solvent B); the proportion of A:B is 70:30. The flow rate was 1.0ml/min. Column eluent was monitored at 254nm to detect emodin, aloe-emodin, rhein, and chrysophanol. Rhein, aloe-emodin, chrysophanol, and emodin peaks were collected. Zaltoprofen was used as an internal standard (IS).

### 2.4. In Vivo Studies with a Rat Model of AP

Male Sprague-Dawley (SD) rats (220 ± 50 g) were maintained under controlled environmental conditions and fasted for 24h with free access to water prior to experiments. All experiments were approved by the Animal Ethics Committee of Sichuan University and carried out in accordance with university guidelines. Pancreatitis was induced in SD rats by 7 intraperitoneal (IP) injections at 1h intervals of 50 *μ*g/kg cerulein, a CCK analogue used for experimental pancreatitis models. Control animals received similar injections of physiologic saline [16]. Stewed rhubarb solution or vehicle was administered by gavage 2h after the model induction. The drug was given every day until the rats were sacrificed. No positive control drug was recommended in the treatment of SAP to date [17]. Rats were randomized into three groups: (1) AP control group, which was given vehicle (0.9% NaCl, 1 mL/kg); (2) AP + stewed rhubarb group (12g/kg, dry herb equivalent); (3) sham-injection control group. The dose of rhubarb in the present study was derived from our previous study in human subjects. We usually give patients rhubarb at 0.3g/kg (dry herb equivalent) per day. That is, the rats were administered approximately 40 times the human dose. Rats were sacrificed on the 1st, 2nd, 5th, and 8th days after cerulein injection. On the day they were sacrificed, the pancreatic microcirculatory blood flow was measured with a laser Doppler flowmeter. Immediately after the measurement of pancreatic blood flow, the abdominal aorta was exposed, and blood was taken for determination of plasma amylase and pancreatic amylase activity, determined by an enzymatic method (amylase reagent, Nanjing Jiancheng, China). At each time point, six rats were sacrificed to obtain sufficient pancreas and other tissue samples for assays. Pancreas were rapidly removed and frozen immediately in liquid nitrogen or fixed for immunohistochemically staining and ultrastructural examination.

### 2.5. Determination of Pancreatic Blood Flow

On the 1st, 2nd, 5th, and 8th days following the injection of saline or cerulein, the animals were anesthetized, and the abdomen was opened. Pancreases were exposed for the measurement of pancreatic blood flow using a laser Doppler flowmeter (PF5010, Sweden). Pancreatic blood flow was measured in five different portions of the pancreas and the area of laser emission of the probe was about 1mm^2^, while the depth of measurement reached about 3mm. These were recorded and presented as the mean perfusion (PU) from five different points (PU).

### 2.6. Determination of Serum Amylase Concentrations

Plasma amylase levels were determined at 37°C by means of an enzymatic assay (Jiancheng, Nangjing, China) with a spectrophotometer. All plasma samples were assayed in duplicate, and the results were averaged at the end of each experiment.

### 2.7. Immunohistochemistry

Regeneration of the damaged pancreas was monitored histologically for the reappearance of zymogen granule-containing acinar cells and the organization of these cells into acini. Replication of pancreas cells was determined by immunohistologic detection of cells expressing Ki-67, as a marker of proliferation, transforming growth factor beta 1 (TGF-*β*1) and actin. Pancreatic tissues were fixed with 4% formaldehyde solution at 4°C for 24 hours, dehydrated in graded concentrations of ethanol, embedded in paraffin, and sliced. The primary antibodies, namely, TGF-*β*1, Ki-67, and actin (abcam, US), were diluted to 1:100. PBS was used as control. For evaluation, the section was scanned at low magnification for the most densely and evenly labeled areas. Unequivocal staining was regarded as a positive reaction, regardless of the staining intensity. Each slice was photographed and the integrated optical density (IOD) was measured with Image pro plus 5.02 (Media Cybernetics, USA).

### 2.8. Ultrastructural Examination

Small specimens (about 1mm^3^) of pancreatic tissue (three from each animal) were immediately fixed in 3.6% glutaraldehyde in 0.1mol/L cacodylate buffer (pH7.4) for 3h and, after washing in the buffer, post fixed in 2% osmium tetroxide for 1h. The samples were dehydrated in alcohol and propylene oxide and then embedded in Epon812. The ultrathin sections were cut from each block on a Reichert ultramicrotome, stained with lead citrate and uranyl acetate, and studied under an Opton900 PC transmission electron microscope field by field. Fifty to sixty electron micrographs of the most characteristic changes from each group were made. The determination of pathology was made blind.

### 2.9. In Vitro Studies with a Cellular Model of AP

The rat pancreatic acinar AR42J cells (ATCC, Rockville, MD, USA) were cultured in F12K medium containing 20% FBS, 100U/mL penicillin, and 100U/mL streptomycin under standard conditions (37°C and 5% CO_2_). AR42J cells (1million/mL) seeded in flat-bottom 6-well plates were divided into the following groups: (1) control group, in which cells were cultured in medium alone; (2) AP model group, in which cells were cultured in medium containing cerulein (10^−8^M, Sigma); (3) treatment group, in which cells were cultured in medium containing cerulein and rhein (1uM), respectively. The concentration of rhein in cell culture is determined by our previous study on plasma and tissue concentrations result of rhein in rats after administration of DCQD [14, 18]. Experiments were performed 24h after cells were seeded. To investigate the protective effects of rhein on regeneration, AR42J cells were pretreated with rhein (1uM) for 30min and were then coincubated with rhein and cerulein for a further 16 h.

### 2.10. Ultrastructural Examination of AR42J Cells

Coincubated with rhein and cerulein for a further 16h, the cells were collected by centrifugation, the supernatant was removed, and the fixed solution was added slowly to fix the 3% glutaraldehyde to maintain the mass of cells. After dehydration and embedding, cells were stained with uranyl acetate and lead citrate and then examined using an electron microscope.

### 2.11. Measurement of Mitochondrial Membrane Potential (MMP)

The effect of rhein on the mitochondrial membrane potential of pancreatic AR42J cells was measured using mitochondrial membrane potential fluorometric assay kits. MMP is a marker of mitochondrial oxidative phosphorylation activity that can be assessed using fluorescent probes accumulating in mitochondria depending on the MMP. For detection of MMP in this study, the fluorescent dye TMRM (tetramethylrhodamine methyl ether, 1uM), which is concentrated by respiring mitochondria, was employed.

### 2.12. Enzyme-Linked Immunosorbent Assays (ELISA)

AR42J cells were resuspended in 1ml complete PBS followed by liquid nitrogen refrigeration. Thawing (6-7 times) until the cells dissolved at 37°C, centrifuged at 3000×g for 10min, the supernatant was collected for cryopreservation. AMPK (Adenosine 5‘-monophosphate activated protein kinase), PI3K (phosphatidylinositol 3-kinase), and AKT (protein kinase B) optical density were measured by ELISA kits (Bassett Weiss Biotech, Shenzhen, China) complying with the manufacturer's protocols on enzyme labeling instrument. The OD value of each sample was determined according to the standard curve and the corresponding curve equation to calculate the concentration of each index in the sample.

### 2.13. Statistical Analysis

Differences in results obtained with stewed rhubarb and rhein treatments were tested for statistical significance using one-way ANOVA. Differences with an associated P value of 0.05 or less were considered significant.

## 3. Results

### 3.1. Analysis of Major Chemical Components in Rhubarb

**Figure 1:**
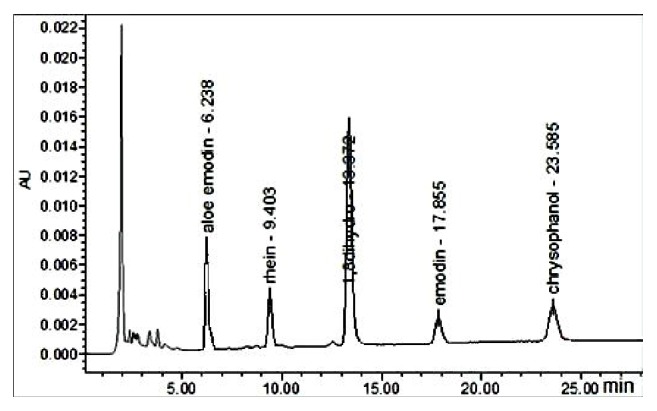
Chromatogram of rhubarb sample emodin, aloe-emodin, rhein, chrysophanol, and internal standards (1,8-dioxyanthraquinone).

Major components of stewed rhubarb include the four anthraquinones with known compounds present in the following relative abundance: emodin > aloe-emodin > rhein > chrysophanol (Figure 1). The following values for content (mg/g) and retention time (min) were obtained for each characteristic compound: emodin, 7.62 ± 1.3, 17.855; aloe-emodin, 5.13 ± 1.08, 6.238; rhein, 2.24 ± 0.34 mg/g, 9.403 min; and chrysophanol, 1.67 ± 0.43, 23.585. Recoveries of all compounds were >95%.

### 3.2. Animal

#### 3.2.1. Plasma Amylase in AP Rats

In AP, inflammation of the pancreatic cells leads to elevated levels of plasma amylase. Plasma amylase was significantly higher in the AP model than that in rats of the sham group. In rats given rhubarb, amylase in serum was lower than that observed in AP rats (P < 0.05) (Table 1).

#### 3.2.2. Pancreatic Tissue Blood Perfusion

**Figure 2:**
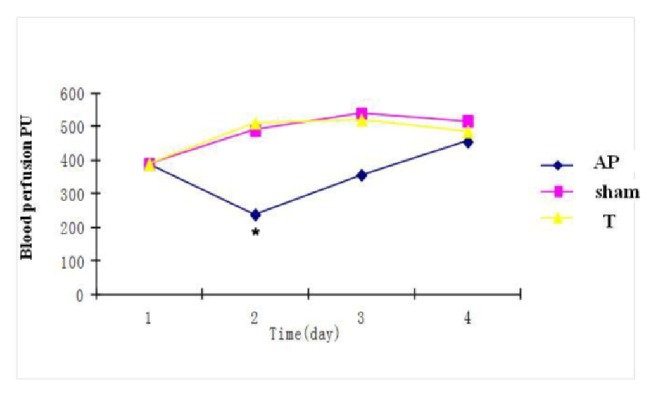
Effect of rhubarb on the pancreatic blood perfusion on different days in three groups. C: control group; AP: acute pancreatitis group; T: rhubarb treated group. *∗*P < 0.05 versus AP group.

The pancreatic tissue perfusion was significantly reduced after the first two days in the AP group. Rhubarb significantly increased pancreatic tissue blood perfusion at day 2 (510 ± 187 versus 230 ± 68). Between day 5 and day 8, pancreatic tissue blood perfusion showed no difference among the normal, AP, and treatment groups (Figure 2).

#### 3.2.3. Acinar Cell Ultrastructure

**Figure 3:**
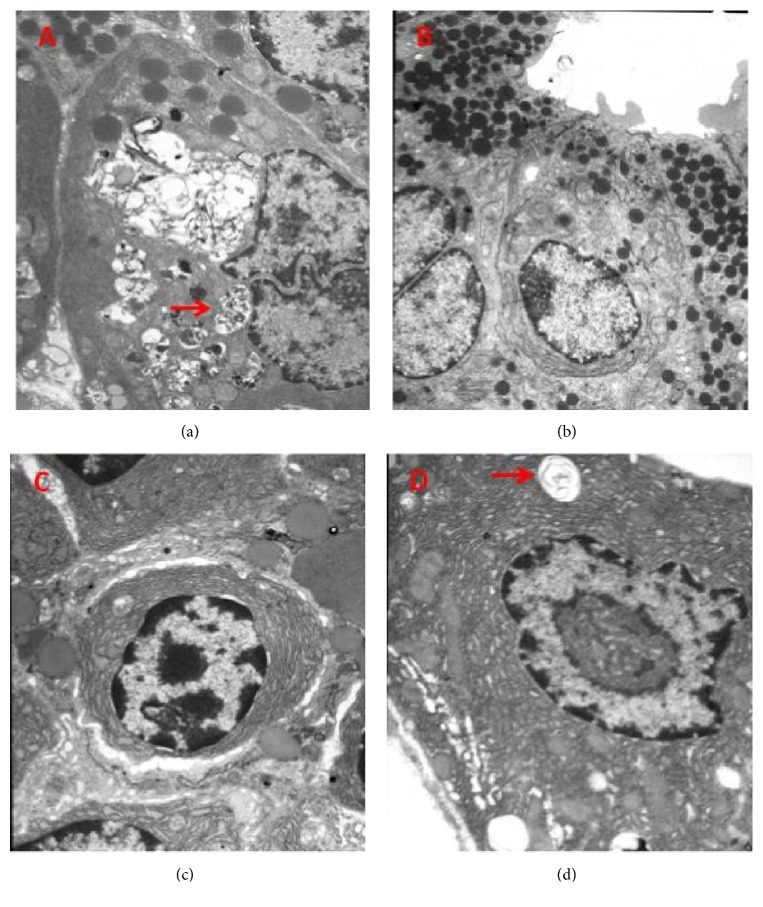
Acinar cell regeneration and ultrastructure were observed from the electron microscopy. (a) In AP rat, there were more lysosomes with more swallowed organelles after pancreas necrosis, rough endoplasmic reticulum mild expansion or disappearance, severe mitochondrial damage where ridges ruptured or disappeared, and mitochondrial swelling (arrows) on the 2nd day. (b) Ultrastructure of acinar cells from the electron microscopy was not detected with obvious morphological changes in the rhubarb treated group on the 2nd day. (c) On Day 5, swelling of mitochondria with a large number of lymphocytes is still found in rhubarb groups. (d) The overall shape of the acinar ultrastructure was ameliorated on the 8th day in the rhubarb treated group. Original magnification: ×1000 (a-d).

Using electron microscopy, we found obvious morphological changes in the model group: more lysosomes with more swallowed organelles after necrosis, rough endoplasmic reticulum mild expansion or disappearance, severe mitochondrial damage where ridges ruptured or disappeared, and mitochondrial swelling on the 2nd day. The rhubarb groups showed a significant reversal of morphology: the intracellular vacuoles decreased or disappeared with a small amount of neutral cells and eosinophils, especially the reversal of mitochondrial morphology from the AP group. On the 5th day, swelling of mitochondria with a large number of lymphocytes was still found in rhubarb groups. However, the overall shape of the acinar ultrastructure showed no difference among all the groups on the 8th day (Figure 3).

#### 3.2.4. Pancreatic Regeneration

**Figure 4:**
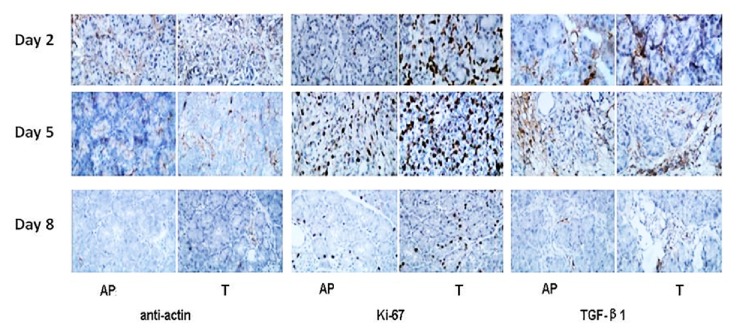
Immunohistochemical analysis of pancreatic tissues of rats for the expression of actin, Ki-67, and transforming growth factor beta1 (TGF-*β*1) on the 2nd, 5th, and 8th days after cerulein-induced AP was induced. Evaluation of photomicrographs of a representative section of pancreatic tissue obtained from either a AP rat or a rhubarb treated rat indicated that there were many more Ki-67, actin, and TGF-*β*1–positive nuclei or matrix detected in pancreatic tissue of rats treated with rhubarb. Arrows indicate Ki-67–positive nuclei of acinar cells. Original magnification: ×200.

The positive actin monoclonal antibody staining reflects stellate cells in the matrix, while Ki-67 staining reflects the pancreatic acinar cell nuclei proliferation. TGF-*β*1, as the most important regulatory factor in extracellular matrix deposition, is a symbol of transient fibroblast proliferation activation. We detected these important tissue regeneration regulatory factors by immunohistochemical analysis. The preliminary results showed that the actin expression was significantly increased and reached a peak on the 2nd day, reduced on the 5th day, and was close to normal on the 8th day; pancreatic cell nuclear proliferating factor Ki-67 and TGF-*β*1 in the extracellular matrix sustained high expression during the 2nd-5th days and decreased to a minimum until the 8th day. The expression of the rhubarb treated group was higher than that of the AP group on the expression peak of each factor (p < 0.05). These results indicate that rhubarb could reduce the pancreatitis pathological damage, promote pancreatic acinar cell and stellate cell proliferation, activate fibroblasts, and promote pancreatic regeneration (Figure 4).

### 3.3. AR42J Cells Study

#### 3.3.1. Mitochondrial Ultrastructure of Exocrine Cells

**Figure 5:**
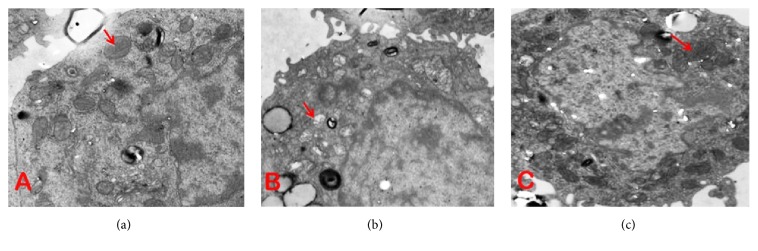
Acinar cell regeneration and ultrastructure were observed from the electron microscopy. (a) AR42J cells: the structures of organelles seemed almost intact. (b) Model group, in which AR42J cells were cultured in medium containing cerulein (10^−8^ M): mitochondria were damaged where ridges ruptured or disappeared (arrows). (c) After 16h of treatment of rhein, mitochondrial swelling and fracture were reduced.

In the model group, in which AR42J cells were cultured in medium containing cerulein (10^−8^ M), more necrosis organelles were contained in the lysosomes after being swallowed. Rough endoplasmic reticulum was dilated or disappeared. Mitochondria swelled and ridges were broken or disappeared, showing vacuolization. After 16h of treatment of rhein, mitochondrial swelling and spinal fracture were reduced. Rhein can significantly improve cell ultrastructure, especially the improvement of mitochondrial morphology (Figure 5).

#### 3.3.2. The Mitochondrial Membrane Potential (MMP)

**Figure 6:**
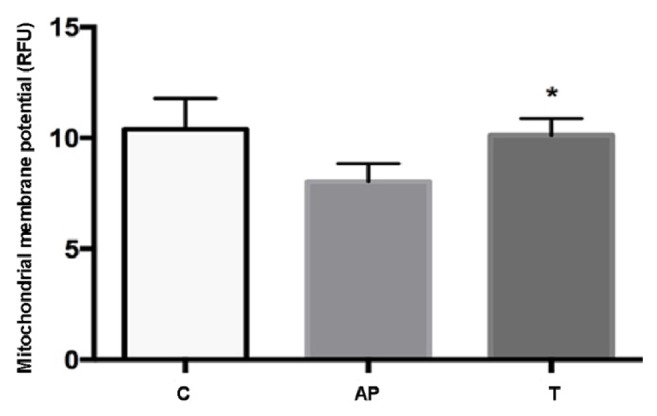
Effect of rhein on the mitochondrial membrane potential of AR42J cells in three groups. C: control group; AP: acute pancreatitis group; T: rhein treated group. *∗*P < 0.05 versus AP group (n = 3).

The fluorescence ELISA data showed that, compared with the control group, cerulein stimulation decreased the mitochondrial membrane potential of AR42J cells, indicating that cerulein damaged the structure and function of mitochondria. After treatment with rhein for 16 hours, the potential of the treated group was obviously restored compared with the model group (P < 0.05), indicating that rhein can effectively lighten the cerulein that caused mitochondrial damage and improve mitochondrial function (Figure 6).

#### 3.3.3. Impact of Rhein on Levels of AMPK, PI3K, AKT, and mTOR in AR42J Cells

**Figure 7:**
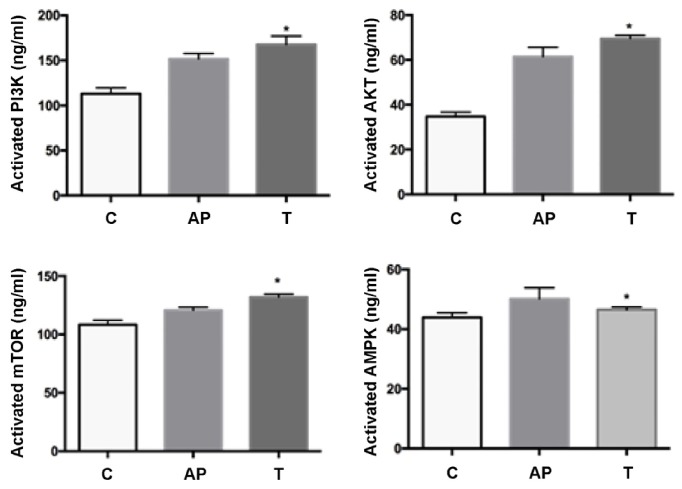
Expression of AMPK, PI3K, AKT, and mTOR was detected using ELISA in three groups. C: control group; AP: acute pancreatitis group; T: rhein treated group. *∗*P < 0.05 versus AP group (n = 3).

Expression of AMPK, PI3K, AKT, and mTOR (mammalian target of rapamycin) was detected using ELISA. As a cellular energy sensor responding to low ATP levels, AMPK activation negatively regulates ATP-consuming biosynthetic processes such as protein synthesis to improve the energy metabolism when pancreatitis happened. The PI3K/AKT/mTOR signaling pathway exerts a significant effect on the regulation of metabolism, cell growth, and survival [19]. Compared with the AP model group, the treatment of rhein (1uM) for 16h increased PI3K, AKT, and mTOR expression in AR42J cells (P < 0.05); while treatment with rhein (1uM) significantly decreased the AMPK expression in these cells (P < 0.05, Figure 7). These results demonstrate that rhein can improve the energy metabolism of pancreatic cells by downregulating the expression AMPK and enhancing the expression of the PI3K/ AKT/ mTOR signaling pathways for protein synthesis.

## 4. Discussion

The clinical sequence and limited pathologic data suggest that the symptoms of mild and severe pancreatitis are comparable in animal models and humans. Both mild and severe forms of the disease have a similar progression and outcome, although its progress is usually more rapid in rodents than in humans [20]. Rhubarb and rhein were chosen for this study based on our previous investigation into the effect of AP on tissue distribution of DCQD and rhubarb individually by HPLC-MS. Using AP rats, we found that rhein has the highest level in the target organ, the pancreas, whether given with DCQD or rhubarb alone. Data from experimental and clinical studies suggest that antibiotics that show good penetration into the pancreas may reduce mortality by preventing pancreatic infection [21, 22]. Whether or not and how much the drug can pass the blood-pancreas barrier and penetrate into tissues (especially the pancreas) may therefore also be the most important factor in the regeneration of the pancreas. Rhein was found to be concentrated in pancreas in the AP model, which suggests it may be the primary active ingredient of rhubarb.

AP is initiated by intracellular activation of pancreatic proenzymes and autodigestion of the pancreas. Destruction of the pancreatic parenchyma first induces a local inflammatory reaction [23], which leads to the dysfunction of microcirculation in the pancreas [24]. We found that treatment with rhubarb was beneficial in cerulein-induced pancreatitis. In AP, serum levels of amylase are known to be elevated [25]. Rhubarb treatment significantly reduced the amylase levels compared to the control group (P < 0.05).

We also observed that induction of AP by cerulein caused an initial reduction of pancreatic blood flow on the 2nd day followed by a subsequent increase in this parameter. Rhubarb caused a reversal of the cerulein-induced fall in pancreatic blood flow in the early stages of the regeneration process. The mechanism of this circulatory effect is likely associated with the well-known anti-inflammatory, blood circulation promotion, and blood stasis removal effects of rhubarb [26]. Previous studies have also shown that an increase in pancreatic blood flow and regeneration may be a cause of improvement of pancreatic disorder, but simultaneously a reduction in pancreatic damage improves pancreatic blood flow [27].

Other experimental studies have shown that administration of growth factors attenuates pancreatic damage and accelerates pancreatic recovery [3, 28]. Transforming growth factor-*β*1 (TGF-*β*1) is one of the most important regulatory factors with the ability to (1) reduce neutrophil adherence to endothelial cells, (2) promote angiogenesis in the pancreas, and (3) stimulate the transient increase in fibroblast proliferation and deposition of extracellular matrix which creates a platform for pancreatic regeneration [29, 30]. In our studies, regeneration parameters of TGF-*β*1, smooth muscle actin positive stellate cells, and ki-67-positive cells all significantly improved with rhubarb treatment between day 2 and day 5, thus indicating the active regeneration process in the pancreas. In our ultrastructural study of pancreatic tissue, rhubarb had a significantly positive effect on the morphology of damaged mitochondria without obviously improving the other organelles' morphology. Mitochondria generate most of the cellular adenosine-5'-triphosphate (ATP) and are very sensitive to the ATP depletion caused by inflammatory reactions occurring in exocrine cells at the initial stage of AP. Mitochondrial injury is therefore assumed to lead to suppression of ATP production and affected inner membrane integrity. The protective effect of rhein on the damaged mitochondrial morphology was also observed in exocrine model cells, with MMP being restored to nearly the normal level in our cell study. These findings provide evidence that rhein contributes to the ability of rhubarb to promote regeneration of the pancreas via mitochondrial protection. In order to further elucidate the underlying mechanism or rhein in driving pancreatic regeneration, regulation of AMPK, PI3K, AKT, and mTOR expression was checked in AR42J acinar cells. It has been demonstrated that decreased activity of AMPK is required for both proliferation and maintenance of the differentiation state in cells [31]. Our data showed that rhein reduced the activity of AMPK in the treatment group (P < 0.05).

Consistent with our results, a study conducted by Halbrook et al. also showed that short-term blockage of AMPK signaling restored exocrine tissue and dramatically reduced fibrosis in established pancreatitis [3]. Moreover, the PI3K/AKT/mTOR signaling pathway is proven to be correlated with neuroprotection and stimulate cell proliferation [32, 33]. Our data demonstrated that rhein upregulated the expression of PI3K, AKT, and mTOR in AR42J cells.

In conclusion, we did in vivo studies of rhubarb and in vitro experiments on one major phytochemical component of rhein from rhubarb to test their effects on pancreatic regeneration. Taking the two sets of results together, rhubarb improved the severity of pancreatitis in cerulein-induced experimental AP in rats and promoted the spontaneous repair and regeneration processes of pancreatic tissue. In addition, the anthraquinone, rhein, had higher penetration into the pancreas in the AP disease state (our previous research), which contributes to the ability of rhubarb to promote regeneration of the pancreas through mitochondrial protection. Our findings are evidence that rhein exerts a pancreatic protective effect via the activation of the PI3K/AKT/mTOR signaling pathway and activates inhibition of AMPK in AR42J cells suffering from cerulein; rhein is therefore a potential new compound for the treatment of AP.

It needs to be stressed that Chinese herbs have many pharmacological substances and therefore have multiple therapeutic effects. Another question is which compound(s) of rhubarb is (are) the most active? A major limitation of the present study is that we conveniently chose the most abundant substance in pancreatic tissue (from rhubarb) to test, but the screening for other possible active compound(s) responsible for regeneration of pancreatic acinar cells was not established in AR42J cells. Future studies utilizing pancreatic AR42J cells to determine the active compound(s) of rhubarb and its mechanism of action may provide more complete answers, and these experiments are being planned.

## Figures and Tables

**Table 1:** Levels of plasma amylase in the three groups (IU/L, m ± SD) (n = 6).

	Sham	AP	Treatment
AMY (IU/L)	992 ± 221	2077 ± 585^*∗*^	1458 ± 450^#^

^*∗*^ *P* < 0.05 versus sham group. ^#^*P* < 0.05 versusAP model group.

## Data Availability

The data used to support the findings of this study are available from the corresponding author upon request.
